# Synbiotics as Supplemental Therapy for the Alleviation of Chemotherapy-Associated Symptoms in Patients with Solid Tumours

**DOI:** 10.3390/nu15071759

**Published:** 2023-04-04

**Authors:** Neeraj K. Singh, Jeffrey M. Beckett, Krishnakumar Kalpurath, Muhammad Ishaq, Tauseef Ahmad, Rajaraman D. Eri

**Affiliations:** 1 School of Health Sciences, University of Tasmania, Newnham, Launceston 7248, Australia; 2 Mersey Community Hospital, Latrobe 7307, Australia; 3 School of Science, STEM College, RMIT University, Melbourne 3083, Australia

**Keywords:** cancer, chemotherapy, probiotics, prebiotics, synbiotics

## Abstract

Chemotherapy is still the first line of treatment for most cancer patients. Patients receiving chemotherapy are generally prone to infections, which result in complications, such as sepsis, mucositis, colitis, and diarrhoea. Several nutritional approaches have been trialled to counter the chemotherapy-associated side effects in cancer patients, but none have yet been approved for routine clinical use. One of the approaches to reduce or avoid chemotherapy-associated complications is to restore the gut microbiota. Gut microbiota is essential for the healthy functioning of the immune system, metabolism, and the regulation of other molecular responses in the body. Chemotherapy erodes the mucosal layer of the gastrointestinal tract and results in the loss of gut microbiota. One of the ways to restore the gut microbiota is through the use of probiotics. Probiotics are the ‘good’ bacteria that may provide health benefits if consumed in appropriate amounts. Some studies have highlighted that the consumption of probiotics in combination with prebiotics, known as synbiotics, may provide better health benefits when compared to probiotics alone. This review discusses the different nutritional approaches that have been studied in an attempt to combat chemotherapy-associated side effects in cancer patients with a particular focus on the use of pre-, pro- and synbiotics.

## 1. Introduction

Cancer is the leading cause of mortality in Australia, leading to approximately 50,000 deaths in 2020. Currently, there are more than 1 million people in Australia who have been diagnosed with cancer at some point in time. It was estimated that around 150,000 new cases would be diagnosed in Australia in 2020 [1]. The World Health Organization (WHO) has estimated that cancer diagnoses will increase by 45% between 2008 and 2030. Major causes of cancer involve heavy smoking, poor diet, physical inactivity, and environmental pollutions. In 2018, the most common cancers diagnosed globally were lung cancer (2.09 million), followed by colorectal (2.09 million), prostate (1.28 million), skin (1.04 million), and then stomach cancer (1.03 million). The financial burden of cancer is enormous and the global burden in 2010 was estimated to be approximately USD 1.16 trillion [2].

Cancer is defined as the uncontrolled growth of cells in the body and, clinically, is termed as a malignant neoplasm. Cancer starts via genetic and epigenetic variations that result in the unlimited multiplication of cells which evade the mechanisms that normally control cell growth and division. This uncontrolled growth and multiplication of cells finally appear as a collection of cells called a tumour. These cells can metastasise to the other parts of the body through the bloodstream or lymphatic system. Cancer treatment depends on the type and stages of the disease [3,4]. Cancer treatment options include chemotherapy, radiotherapy, surgery, immunotherapy, and monoclonal antibody therapy [5]. Despite recent advances in cancer treatments, chemotherapy is still a cornerstone of cancer therapy [5,6].

## 2. Cancer Chemotherapy

The term “chemotherapy” was coined by Paul Ehrlich in the early 1900s for drug therapy in the treatment of diseases. He also documented the efficacy of certain chemicals against diseases in animal models, which later led to the development of cancer drugs. Chemotherapy was first introduced in cancer treatment in the 1940s and 1950s [7]. Chemotherapy is an essential part of cancer treatment, and the development of new anticancer drugs represents one of the major areas in pharmaceutical research [8]. Chemotherapy is often the only option for the oncologist when cancer has widely metastasised to other parts of the body [9]. The major disadvantage of chemotherapy is unwanted cytotoxicity, as it cannot discriminate between rapidly dividing cancer cells and normal cells undergoing cell division [10]. However, cancer cells are generally more sensitive to the cytotoxic action of chemotherapy agents when compared to normal cells. A combination of drugs used at regular intervals can cure some cancers while others can be palliatively managed in order to improve the patient’s symptoms and quality of life [3,11]. The main aim of chemotherapy is to reduce the cancer cell population to a minimal level. The fractional cell kill hypothesis, primarily offered for haematological and lymphatic malignancies, has been accepted as the protocol for various other cancer types, including solid tumours. As per this hypothesis, a specific concentration of drug in a defined period will kill a constant number of the cell population, irrespective of the absolute number of tumour cells [12,13]. The treatment efficacy depends on the dose of drug, as well as on the number and frequency of chemotherapy cycles as each successive cycle of chemotherapy will eliminate only a fixed number of remaining cells [14]. Hypothetically, a tumour size of 10^11^ cells will be reduced to less than one cell after six cycles of chemotherapy, if 99% of the cells are killed per cycle [15]. The ability of normal tissues, such as in bone marrow and the gastrointestinal tract, to recover after chemotherapy decides the timing of chemotherapy cycles; this is usually about three to four weeks [15,16].

The majority of chemotherapeutic drugs target dividing cells and thus are more effective in tumours with rapidly dividing cells. Some drugs act on a specific phase of the cell cycle in dividing cells, while a few target only the non-dividing cells. A sudden decrease in tumour size by surgery (debulking), radiotherapy, or chemotherapy induces cell division and consequently increases the susceptibility of the tumour to chemotherapy.

Currently, a newer anticancer therapy, known as targeted therapy, is also widely in use. The goal of targeted therapy is to deliver the drug to specific molecules of interest in cancer cells or in the tissue environment, thereby regulating the growth and development of the cancer. This molecule-specific action of treatment has been shown to be beneficial in many cancers and is now used globally [10,17]. Monoclonal antibodies (immunotherapy) and small molecule inhibitors (cellular kinases) are the two main categories of targeted therapy. Monoclonal antibodies induce cytotoxicity by different mechanisms, such as target cell killing through the recruitment of host immune functions, by receptor or ligand binding, to disturb the essential cancer cell processes or by deadly payloads, such as radioisotopes or toxins to kill the target cells. They are administered intravenously and circumvent the first pass/hepatic metabolism [17,18]. On the other hand, small molecule tyrosine kinase inhibitors (SM-TKIs) are orally administered and known to inhibit oncological targets in many solid organ tumours. Unlike conventional chemotherapy, the SM-TKIs, which include VEGFRs (vascular endothelial growth factor receptors) TKIs, and EGFRs (epidermal growth factor receptors), TKIs can be administered orally for a longer duration (i.e., months or years) [19]. Targeted therapy also exerts side effects as it damages the normal cells that express target molecules. However, side effects in this case can also be used as surrogate markers of the treatment efficacy [10,18].

Cancer treatment generally uses a combination of chemotherapy to reduce toxicity and to reduce the risk of resistance against the drugs [15]. The majority of cancer patients undergo conventional chemotherapy treatment; however, this is associated with many complications including widespread mucositis, which can manifest as pain, inflammation, bleeding, risk of infections, and diarrhoea [20].

### Chemotherapeutic Agents and Side Effects

The toxicity of chemotherapy is a major cause of concern, leading to a poor quality of life in cancer patients and may eventually result in a reduction in dose in order to manage the adverse effects of the treatment. It has been shown that reduction in dose results in low survival rates [8]. Currently, a wide range of chemotherapeutic agents are in use and exhibit a variety of side effects in cancer patients. The different classes of chemotherapy drugs, based on their mechanism of actions and side effects, are summarised below in Table 1.

It is clear from the above discussion that chemotherapy, while an important part of cancer treatment, is associated with numerous adverse effects in patients. Several approaches have been used to reduce the chemotherapy-induced side effects, but they have not been fully effective. Recent studies have reported on the beneficial effect of gut microbiota on cancer and on chemotherapy side effects. Our understanding of the importance of the gut microbiota is still developing but given the prominence of gastrointestinal symptoms for many of the chemotherapeutic agents, this review will explore the efficacy of maintaining or manipulating the gut microbiota on cancer and on chemotherapy-associated side effects.

## 3. Gut Microbiota

The human body contains 10–100 trillion microorganisms, including bacteria, fungi, archaea, and viruses. The majority of these microorganisms live in the human gut, mostly in the large intestine, and are collectively referred to as the gut microbiota. The gut generally hosts a heterogeneous population of about 1000 bacterial species. Recent studies have recognised that gut microbiota play a pivotal role in maintaining the host’s metabolism, immunity, and overall wellbeing [31,32,33,34].

The gastrointestinal system is the connecting link, as well as the barrier between the gut microbiota and the major organs in the human body. It secretes essential hormones that play crucial role in functions, such as neuromodulation, digestion, and gastrointestinal motility. Gastrointestinal hormonal secretions vary according to the body’s internal environment and in response to psychological or physiological stresses. It has been shown that variation in hormonal secretions can alter the composition of gut microbiota [35,36,37]. On the other hand, gut microbes generate or convert bioactive molecules into forms that can be recognised by the gastrointestinal cells and, in turn, promote immunomodulation, metabolic modulation, protection from gut pathogens, xenobiotic metabolism, and the maintenance of gut barrier integrity [32,38]. Some bacterial genera, such as *Bifidobacterium, Bacteroides*, and *Enterococcus*, which are found in the gut, are known to synthesise important micronutrients, such as vitamin K, vitamin B, and signalling molecules by converting glutamate into gamma-amino butyric acid (GABA) or histidine to histamine. In addition, gut commensal bacteria also transform inactive complex polyphenols and primary bile acids into their absorptive active phenolic compounds and secondary bile acids, respectively [32,39,40,41]. Gut microbiota also produce conjugate linoleic acid, which is known to have an anti-diabetic property [32]. Furthermore, the gut microbiota fermentation of dietary fibres generates short-chain fatty acids (SCFAs). SCFAs are not only used as an important source of energy for the cells lining the colon, but also control the metabolism of carbohydrates and lipids by affecting the epithelial cell secretions in the gut [38,42].

As there is a bidirectional communication between the gut microbiota and its host, the maintenance of adequate composition and the number of the gut microbial population is essential for the appropriate regulation of the host’s key metabolic and immune functions [43,44,45]. Any alteration in this crucial balance may cause dysbiosis, a condition that is associated with many human diseases, including cancer [46,47].

The gut microbiota, with its whole genome referred to as the gut microbiome, encodes over 100 times more genomic information than the human genome [48,49]. Metagenomics studies have enabled researchers to characterise the diversity and richness of the gut microbiome, with the aim to determine the effect of individual gut microbial species on the host. Over the last decade, researchers have studied faecal microbial cultures using metagenomic evaluation techniques, such as next-generation sequencing (NGS) and bioinformatic tools for the analysis of the 16S rRNA amplicons, as well as shotgun metagenomics for the profiling of microbes [50,51,52]. These techniques reveal the profound influence of microbiome diversity and its composition on human health, as is shown by the Human Microbiome Project [53,54,55,56].

## 4. Modulation of Gut Microbiota

The gut microbial environment can be influenced and repopulated with beneficial bacteria, specifically with the judicious consumption of a combination of probiotic, prebiotic, and synbiotic formulations [57].

### 4.1. Probiotics

Probiotics are living microorganisms that confer benefits to human host health when administered in adequate amounts [58]. In the early 1900s, Elie Metchnikoff was the first to postulate that human wellbeing can be enhanced by modifying the gut microbial composition with beneficial microbes. With the advancement of the knowledge on probiotics, it is now recognized that probiotics not only influence the repopulation of the gut microbiota, but also stimulate the physiological and metabolic changes in the host [59]. Yoghurt and fermented food contain many naturally occurring bacteria, which could also be considered probiotics. Bacterial species considered to be probiotics include a variety of microorganisms, most particularly *Lactobacillus* and *Bifidobacterium* bacteria, as well as non-pathogenic yeasts, including *Saccharomyces boulardii* [57].

Probiotic use is recommended to control microbe-related gut dysbiosis and to restore and maintain balance in gut microbiota by adhering to host tissue and limiting the colonisation by pathogenic microbes. Several studies have shown that the consumption of certain probiotics reduces the colonisation of pathogenic microbes, including *Clostridium difficile* and *Staphylococcus aureus*, thus supporting the importance of probiotics to avoid microbial infections in the gut [60,61]. Probiotics prevent and/or reduce non-beneficial colonisation in the gut microbial constitution through nutrient competition and surface adherence on epithelial cells or in the mucus, or instead by outnumbering the pathogen colonisation [59]. Probiotics also produce bacteriocins or metabolites, such as acetic and lactic acid that inhibit the growth of pathogens by antimicrobial activity and by pH alteration, respectively [62,63].

Probiotics may induce an immunomodulatory effect that can reduce colonic inflammation or enhance immunosurveillance, depending on the capacity of individual probiotic strains [64]. Probiotics, such as *Bifidobacterium infantis* and *Bifidobacterium breve*, can activate intestinal dendritic cells by interacting with Toll-like receptors and inducing retinoid acid metabolism [65,66]. This activation of dendritic cells leads to expression of type 1 regulatory T cells, Foxp3+ regulatory T cells, and IL-10 release. Conversely, some probiotic strains may exert a proinflammatory-mediated immune response by stimulating increased natural killer cell activity and phagocytotic competence in order to remove infectious pathogens [67,68].

Probiotics also produce beneficial effects on the gut mucosa by strengthening gut barrier integrity. They increase butyrate production, which is used as a substrate for energy by the gut cells and leads to an enhanced expression of tight junction proteins [69]. Probiotic strains, such as *Lactobacillus rhamnosus*, *Lactobacillus plantarum* and *Escherichia coli Nissle* 1917, improve the gut barrier function by promoting the expression of tight junction proteins, such as claudin-1 and occludin, whereby mucin production is stimulated, thus reducing inflammation and enhancing epithelial restoration [59,70,71].

### 4.2. Prebiotics

Gibson and Roberfroid defined the concept of prebiotics in 1995 as a nondigestible food constituent that selectively promotes beneficial bacterial growth, activity in the gut, and the provision of better health [72]. The term non-digestible food constituents generally indicate only conventional carbohydrate- and fibre-based prebiotics, whereas other substances, such as polyunsaturated fatty acids and polyphenols, have also been suggested to have prebiotic potential over the last decade. Thus, all together, prebiotics have been defined as a substrate that is selectively used by gut microbes to confer health benefits to the host [73].

The effects of prebiotics on specific probiotics were first examined using culture-based models with the *Bifidobacterium* and *Lactobacillus* species [74]. However, the advent of high-throughput sequencing technology has greatly enhanced the ability to understand the effects of prebiotics on other gut microbes. Research studies have found that the administration of prebiotics has enhanced the abundance of beneficial bacteria, such as the *Akkermansia*, *Ruminococcus*, *Faecalibacterium* and *Rosebura* species in the gut [75,76,77]. Clinical studies have also shown significant decreases in the colonisation by pathogens and the inflammatory response in patients with chronic intestinal inflammation, after the consumption of prebiotics [78].

As discussed, the gut bacterial fermentation of prebiotics produces SCFAs, such as acetate, propionate, and butyrate. Butyrate is generally utilised by colonocytes as a source of energy, whereas propionate and acetate are taken up by the liver and by muscles for the generation of glucose and energy, respectively [79]. As mentioned previously, butyrate has been shown to enhance epithelial barrier function [80]. Propionate and acetate have also been reported to reduce colonic inflammation and reduce the rates of gut infection [81,82].

Prebiotics may also act directly on the gut and produce an anti-adhesive effect against pathogens. Prebiotic oligosaccharides possess a similar structure to microvillus glycoconjugates and can interact selectively with the pathogenic bacterial receptor in order to prevent their attachment to gut epithelial cells, thus inhibiting pathogen colonisation [83,84,85]. Prebiotics are also thought to be directly taken up by intestinal cells and can modulate gene expression. One animal study has shown that prebiotics with low degrees of polymerisation can increase the production of IFN-γ and IL-10 in CD4+ T cells, thereby suggesting its intact absorption in the intestine and, consequently, a change in the intestinal immune response [86].

### 4.3. Synbiotics

Probiotics are often consumed in combination with a prebiotic; furthermore, such a mixture is known as a synbiotic [87,88,89]. The use of synbiotic preparations is considered to improve the survival of the constituent beneficial microorganisms during passage through the gut by enhancing bacterial resistance against unfavourable environmental conditions, such as adverse temperature, pH, and oxygenation. In addition, it may also support the growth of other native gastrointestinal bacterial strains [90,91,92]. Synbiotic combinations therefore appear to be more efficacious than either the administration of probiotics or prebiotics alone; however, the mechanism of action of their constituents within the body remains the same.

Synbiotics promote an increased modulation of metabolic activity in the gut with improved intestinal integrity, immune regulation, a growth of beneficial microbes, and the greater fermentation of fibres to release SCFAs when compared to the administration of probiotics and prebiotics alone [93]. Additionally, according to a recent study, synbiotic consumption was found to reduce the accumulation of unwanted metabolites, nitrosamines, and carcinogenic substances, as well as up-regulating the production of certain substances, such as carbon disulphides, methyl acetates, and ketones [94].

Clinical data show synbiotics appear to be effective in reducing the severity of specific pathological conditions in the gut. One recent meta-analysis of five studies in children with acute diarrhoea found that synbiotic supplementation was more effective in reducing diarrhoea and hospitalization when compared to probiotic supplementation alone [95]. A study investigating the effect of a synbiotic containing different types of probiotics, such as *Lactobacillus acidophilus*, *Lactobacillus plantarum*, *Lactobacillus delbrueckii* spp. *bulgaricus*, *Lactobacillus rhamnosus*, *Bifidobacterium bifidum*, and inulin as a prebiotic, in non-alcoholic steatohepatitis patients, showed a marked decrease in intrahepatic triacylglycerol (IHTG) within six months [96]. A subsequent clinical study on 52 patients with non-alcoholic fatty liver disease (NAFLD) found that synbiotic supplementation results in the inhibition of nuclear factor-*κB* (NF-κB) and a decreased production of TNF [97], thus indicating that the supplementation with synbiotics was associated with a reduction in inflammation.

## 5. Synbiotic Therapy to Alleviate Chemotherapy-Associated Symptoms

The main side effects of chemotherapy and radiotherapy related to the gastrointestinal system are gut dysbiosis and mucositis. These manifest as painful mouth and oesophageal ulcers, as well as the development of abdominal pain and diarrhoea, thus leading to dehydration and malnutrition in patients with solid organ tumours. Several nutritional approaches—such as prebiotics, probiotics, and, recently, their combination as synbiotics—have been used to enhance the gut microbiota and to minimise the side effects of anti-cancer therapies.

### 5.1. Effect of Prebiotics

*β*-glucans are soluble fibres that consist of biologically active polysaccharides and are generally obtained from bacteria, fungi, and plant sources. *β*-glucan has been reported to have biological properties, including anticancer, anti-inflammatory, and immunomodulating activities [98]. It has also shown prebiotic effects when used in combination with probiotics due to its beneficial effect on probiotic metabolism and growth [99]. *β*-glucan that is obtained from oats has been shown to induce the in vitro growth of *Lactobacillus* and *Bifidobacterium* [100]. A clinical study demonstrated that the use of *β-*glucan-rich durum wheat flour and whole-grain barley pasta not only can increase the population of beneficial microbes such as *Ruminococcus* sp., *Clostridium orbiscindens*, and *Clostridium* sp., but can also decrease the number of *Firmicutes* and *Fusobacteria* in the gut [101]. Another study, using *β*-glucan in 62 patients with colorectal cancer, showed no significant decrease in leucocyte and neutrophil cell counts when compared to the administration of chemotherapy alone during FOLFOX-4 treatment. In addition, *β*-glucan was also able to reduce the incidence of diarrhoea and oral mucositis [102]. Although leukocyte and neutrophils cell counts did not decrease in the *β*-glucan-receiving group undergoing chemotherapy, it is difficult to interpret its beneficial effect on the efficacy of chemotherapy in eliminating solid tumour/cancer cells.

Honey has been used to treat digestive ailments since ancient times. Some honey types possess antibacterial and anti-inflammatory activities; furthermore, they can also promote wound healing [103]. Honey includes non-digestible oligosaccharides and studies have suggested that certain types of honey can act as a prebiotic by which to enhance the beneficial microbial population, including *Bifidobacterium* spp. and *Lactobacillus* spp., in the gut. This enhanced microbial population can help to relieve the symptoms of constipation and ulcerative colitis [103,104,105,106,107]. A Cochrane review of three studies investigating honey suggested that it was able to provide mild-to-moderate reduction in radiotherapy-induced oral mucositis [108]. Cho and colleagues also found that the oral administration of honey was effective in preventing the development of radiotherapy-induced moderate-to-severe oral mucositis and its associated weight loss [109]. Another study conducted by Xu et al. found that honey treatment could reduce the chemoradiotherapy-induced incidence of oral mucositis when compared to no treatment [110]. Honey administration was also able to reduce treatment interruptions, weight loss, and to delay the incidence of oral mucositis. However, it did not decrease the severity of the mucositis grade [111].

### 5.2. Effect of Probiotics

The enrichment of the gut microbiome through the oral administration of probiotics has been used to reduce the adverse effects of chemotherapy, as well as to decrease the chemotherapy-induced gastrointestinal side effects, such as diarrhoea and mucositis [112,113]. Generally, the administration of probiotics in clinical settings are known to have a wide range of benefits, including the improvement of antibiotic- and *Clostridium*-*difficile*-related diarrhoea, as well as the improvement of respiratory tract infections [114]. The administration of probiotics in cancer patients re-establishes the abundance and the functionality of the commensal gut bacteria, which has been affected by anticancer treatment [115]. The major concerns related to using probiotics in immunosuppressed cancer patients are regarding the opportunistic infections and development of antibiotic resistance [38]. Probiotics are live microbes and hence may increase the risk of potential infection in the setting of compromised immunity. However, it has been shown that the administration of probiotics has re-adjusted the composition of healthy gut microbiota with improvements in diarrhoea and other treatment-related damage, including mucositis [113]. The mechanisms in which probiotics may be of benefit in chemotherapy is summarised in Table 2. Probiotic supplements comprising the *Lactobacillus* species have been recommended for the prevention of chemotherapy- and/or radiotherapy-induced diarrhoea and mucositis in patients with pelvic malignancy [38,116]. Several research studies are currently exploring the therapeutic effect of gut microbiota alteration by administering probiotics as food supplements in cancer patients, along with their chemotherapy or radiotherapy. These ongoing research studies point towards the great therapeutic potential of probiotics. A randomised double-blinded clinical trial was conducted in 2010 in cancer patients, who had undergone colorectal resection, and found that the administration of probiotics was beneficial to the composition of gut microbiota and to the regulation of intestinal immune functions [117]. Specifically, *Lactobacillus johnsonii* was able to reduce the concentration of gut pathogens and to modulate local immunity by adhering to the colonic mucosa [117]. In 2014, a clinical study administered the probiotics *Lactobacillus acidophilus* and *Bifidobacterium longum* in patients with pelvic malignancy and reported that 35% of the patients in the group using probiotics did not experience radiotherapy-induced moderate or severe diarrhoea when compared to only 17% in the placebo group [118]. Furthermore, in 2015, a clinical study investigated the safety and efficacy of a probiotic formulation comprising multiple bacterial strains, including *Lactobacilli* and *Bifidobacteria*, in patients with colorectal cancers who were receiving irinotecan-based chemotherapy. The study showed a decrease in the overall incidence of diarrhoea in patients receiving probiotics (39.1%) when compared to the placebo group (60.9%). Enterocolitis was not reported in the probiotic group when compared to 8.7% in the placebo group in a study conducted in patients receiving chemotherapy [118]. A randomised clinical study using *Saccharomyces bulardii* in patients with colorectal cancer undergoing colon resection reported significant down-regulations of pro- and anti-inflammatory cytokines in the intestinal mucosa [119].

Clinical studies using probiotics to reduce the incidence of chemotherapy or radiotherapy-induced intestinal symptoms have shown inconsistent results [120]. They have measured different parameters, such as the frequency and consistency of loose stools, the use of drugs to control diarrhoea, and the change in gut microbiota due to chemotherapy or radiotherapy. However, probiotic intervention appears to be beneficial in the prevention of radiotherapy- or chemotherapy-induced gut toxicity without any significant side effects. As per the Multinational Association of Supportive Care in Cancer’s and the International Society of Oral Oncology’s (MASCC/ISOO) guidelines, probiotics, including the *Lactobacillus* species, can be used to help avoid diarrhoea in patients undergoing chemotherapy or radiotherapy for pelvic cancer [121]. However, the European Society for Clinical Nutrition and Metabolism’s (ESPEN) guidelines suggest that there is not enough clinical evidence to confirm probiotics should be used to reduce radiotherapy-induced diarrhoea [122].

**Table 2:** The beneficial mechanisms of probiotics and their relevance to chemotherapy.

Beneficial Mechanism of Probiotics	Type of Probiotics	Relevance to Chemotherapy	References
The colonization and normalization of dysbiotic gut microbiota	*Bifidobacterium*, *Lactobacillus reuteri*, *Lactobacillus rhamnosus GG*, *Butyricicoccus pullicaecorum, Faecalibacterium prausnitzii, Roseburia hominis, Eubacterium hallii*, and *Anaerostipes caccae*	Chemotherapy may cause the dysbiosis of gut microbiota. Probiotics have been reported to be helpful in re-establishing the microbial communities in the gut. This has been found to be efficient in reducing the chemotherapy-associated gastrointestinal side effects, such as mucositis and diarrhoea.	[38]
Bacterial competition	*Bifidobacterium* and *Lactobacillus*	The depletion of gut microbiota due to chemotherapy results in the abundance of pathogenic bacteria in the gut. Probiotic consumption can outnumber the pathogenic bacteria by bacterial competition and thus reduced chemotherapy-associated side effects.	[38,123]
Cell adhesion	*Lactobacillus rhamnosus*, *Lactobacillus plantarum*, and *Lactobacillus johnsonii*	Chemotherapy damages the gut mucosa and results in the loss of gut microbiota. Probiotics possess the property of adherence and hence can adhere to mucosa in order to enhance the population of beneficial microbes in the gut.	[113]
Intestinal barrier integrity	*Escherichia coli Nissle 1917, Lactobacillus reuteri, *Lactobacillus rhamnosus* GG*, and *Lactobacillus plantarum*	Chemotherapy causes the impairment of the intestinal barrier. The maintenance of the intestinal barrier is the key to control dysbiosis and thus septic infections. Probiotics help to strengthen the integrity of the intestinal barrier.	[124,125]
The modulation of the immune system	*Lactobacillus salivarius, Lactobacillus casei Shirota, Lactobacillus rhamnosus, Lactobacillus casei, Lactobacillus plantarum, Lactobacillus fermentum, Lactobacillus acidophilus, Streptococcus thermophilus, Bifidobacterium breve*, and *Bifidobacterium bifidum*	Chemotherapy may weaken the immune system and compromise its ability to fight against infection. Probiotics regulate the immune response by modulating the functions of immune cells, such as macrophages, dendritic cells, as well as T and B lymphocytes.	[126,127]

### 5.3. The Effect of Synbiotics

Research is now beginning to focus on investigating the effect of synbiotics on anticancer treatment-induced symptoms. Recently, synbiotic (a combination of *Bacillus coagulans* and prebiotic sugar cane flour) administration in an IBD mouse model in our laboratory has shown significant reduction in disease severity, colonic mucosal damage, and inflammation [93]. Another study used a synbiotic formulation containing probiotic *Lactobacillus fermentum* and the prebiotic fructo-oligosaccharide (FOS) in 5-fluorouracil-injected rats and found reduced treatment-induced inflammation in the small intestine [128]. In 2016, a clinical study found that the use of synbiotics—composed of the probiotics *Pediococcus pentosaceus*, *Leuconostoc mesenteroides*, *Lactobacillus paracasei* ssp. *paracasei* 19, and *Lactobacillus plantarum*, as well as the prebiotics *β-*glucan, inulin, pectin, and resistant starch—was able to reduce the risk of developing postoperative complications, such as irritable bowel syndrome (IBS) in cancer patients undergoing colorectal cancer resection [129]. Subsequently in 2017, another clinical study showed that the perioperative use of a synbiotic formulation—containing the probiotics *Lactobacillus acidophilus*, *Lactobacillus rhamnosus*, *Lactobacillus paracasei*, and *Bifidobacterium lactis*, as well as the prebiotic FOS—can significantly reduce the rates of post-operative infection in colorectal cancer patients [130]. Moreover, in 2018, a clinical study observed that the preoperative administration of a synbiotic formulation consisting of *Lactobacillus acidophilus, Lactobacillus rhamnosus, Lactobacillus casei, Bifidobacterium lactis*, and FOS for seven days in patients with colorectal cancer was associated with reduced inflammation, morbidity, use of antibiotics, and reduced length of hospital stay [131]. The effect of synbiotic administration in patients scheduled to undergo colorectal surgery has also been investigated in a randomized clinical trial (*n* = 73) [132]. The patients were randomised into three groups, including a prebiotics group (received prebiotics only), a synbiotics group (received synbiotics only), and a third group that underwent preoperative mechanical bowel cleansing. No significant differences in the systemic inflammatory response were observed after colorectal surgery. However, more lactic acid-producing bacteria were noted in the synbiotic group when compared to the other groups, indicating synbiotic use may have still had a beneficial effect on the gut microbiota.

**Figure 1:**
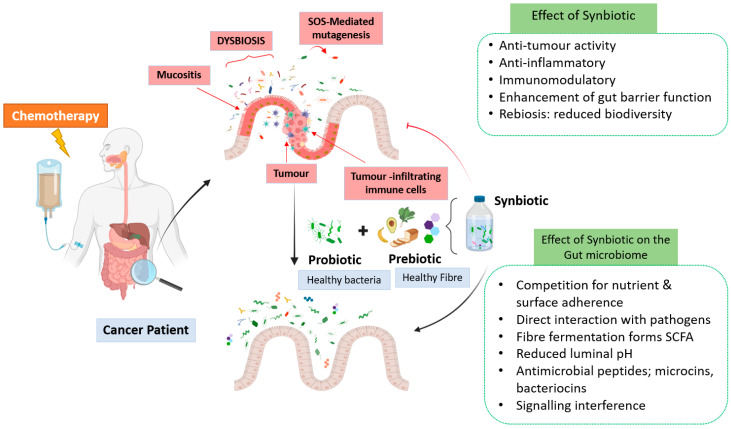
Cancer and chemotherapy may deplete gut microbiota and thus lead to a development of mucositis and the dysbiotic condition. Synbiotic administration may help to restore the dysbiotic gut microbiota that were lost due to cancer and chemotherapy. A schematic representation of an unhealthy gut due to cancer and chemotherapy (on **top**) and a healthy restored gut microbiota (in **bottom**) after synbiotic administration during chemotherapy is shown.

As discussed, patients receiving chemotherapy also develop complications such as colonic infections, mucositis, and diarrhoea. These symptoms may be alleviated by the use of synbiotics through various mechanisms, as shown in Figure 1. One clinical study in 2017 investigated the effect of synbiotics in patients (*n* = 61) receiving neoadjuvant chemotherapy for oesophageal cancer; it was reported that a significant reduction in chemotherapy-induced lymphopenia and diarrhoea in patients using synbiotics was observed [133]. In 2020, another clinical study demonstrated the effect of synbiotic supplementation on colorectal cancer patients (*n* = 46) undergoing chemotherapy. The study reported a slight decrease in the mean symptom score for diarrhoea in the synbiotic group when compared to the placebo group where it increased significantly [134]. More recently, a randomised clinical trial found that the administration of synbiotics prevented bacteremia and reduced gastrointestinal toxicities, including diarrhoea in oesophageal cancer patients (*n* = 42), for those receiving chemotherapy [135]. Even though synbiotic supplementation seems to reduce chemotherapy-induced lymphopenia, bacteremia, and diarrhoea symptoms, it is difficult to be conclusive due to the limited number of studies. Complicating matters further, these studies used different synbiotic combinations, outcome measures, dose interventions, treatment durations, and sample sizes. Therefore, further well-designed clinical studies are required to understand the appropriate dose, duration of supplementation, and the interplay between the administration of chemotherapy regimens and the effect of synbiotics on chemotherapy-induced complications, such as mucositis and diarrhoea. This will help the development of evidence-based microbiota-associated interventions in this cohort.

## 6. Conclusions

Cancer patients undergoing chemotherapy are generally susceptible to infections that may result in various complications, such as sepsis, organ failure, or gastrointestinal tract disruption, including mucositis, colitis, and diarrhoea. This may result in hospitalisation, the discontinuation of chemotherapy, and poor survival in this cohort. Numerous approaches have been studied to avoid such chemotherapy side effects, but they are not yet approved for clinical use or chemotherapy management protocols. This is due to either the trials being underpowered or the results not achieving clinical and/or statistical significance. One of the approaches recently gaining momentum to minimise or avoid the chemotherapy side effects is by the prevention of gut dysbiosis and the repair of gut mucosa in cancer patients. It is hypothesised that this can be achieved through the manipulation of gut microbiota.

Gut microbiota are essential for healthy gut function; however, their composition can be negatively affected by disease treatments, including antibiotics and chemotherapy. Chemotherapy causes serious damage to the intestinal mucosal layer and results in a change in composition and a loss of beneficial gut microbiota. This depletion leads to the development of gut dysbiosis. Therefore, current research efforts are aiming towards the development of approaches that can be used to safely restore gut mucosal integrity and reduce dysbiosis. This should assist in alleviating the detrimental gastrointestinal side effects of chemotherapy, radiation therapy, and immunotherapy, such as mucositis and diarrhoea.

The use of probiotics during anticancer therapy is showing encouraging clinical outcomes by better maintenance of microbial equilibrium in the gut. Patients consuming probiotics during their chemotherapy cycle have shown less gastrointestinal side effects, including diarrhoea and mucositis, when compared to those without probiotics. Due to reduced gastrointestinal side effects, probiotic use is associated with significantly enhanced patient compliance to treatments, thus improving overall quality of life and prognosis. The efficacy of probiotics can be enhanced by co-administration with prebiotics and this combination is known as synbiotics. This synergistic combination provides a better survival rate of probiotics in the gut environment; however, its efficacy in reducing chemotherapy-associated side effects has not yet been well explored. Therefore, future studies should focus on well-designed human trials to study the efficacy of synbiotics in patients undergoing radiation, as well as in targeted and conventional cancer chemotherapy.

## Figures and Tables

**Table 1:** Classes of chemotherapy drugs, drug names, their mechanisms of action, and their common side effects, with references.

Drug Class	Drug Names	Mechanism of Action	Common Side Effects	References
Tubulin modifying agents	Docetaxel and paclitaxel	Inhibit the mitotic process of cells by interfering with the tubulin polymerisation process in order to induce cell death.	Ischaemic colitis, nausea, fatigue, flushing, fever, diarrhoea, acute abdominal pain, neutropenia, septicaemia, hyperglycaemia, gastrointestinal haemorrhage, bowel perforation, neuropathy, dyspnoea, peritonitis, and tenderness.	[21,22]
Platinum-based drugs	Cisplatin and oxaliplatin	Cause DNA damage to induce cell death.	Nausea, vomiting, diarrhoea, constipation, stomatitis, gastro-oesophageal reflux, anorexia, cachexia, asthenia, melena, dry mouth, gum inflammation, haemoptysis, colitis, ileus, pancreatitis, hepatic sinusoidal dilatation, rectal haemorrhage, haemorrhoids, tenesmus renal and neural toxicity, cardiotoxicity, ototoxicity, alopecia, and bone marrow suppression.	[23,24,25]
DNA intercalator drugs	Anthracyclines, doxorubicin, daunorubicin, idarubicin, and epirubicin	Inhibit DNA isomerase II and DNA replication to cause cell death.	Cardiac toxicity, nausea, vomiting, stomatitis, oesophageal ulceration, colonic ulceration, anorexia, and rarely tongue hyperpigmentation.	[10]
Antimetabolites	5-fluorouracil, capecitabine, 6-mercaptopurine, cytarabine, gemcitabine, and methotrexate	Induce cell death during the S-phase of the cell cycle or by inhibiting the enzymes responsible for nucleic acid production	Fever, nausea, vomiting, gingivitis, pharyngitis, gastrointestinal ulceration, abdominal pain, loss of appetite, haematemesis, melena, diarrhoea, constipation, stomatitis, bowel necrosis, pancreatitis, hyperbilirubinemia. hepatic failure, hyperbilirubinemia, dyspepsia, anorexia, bone marrow suppression, and leukopenia.	[10,26,27]
Alkylating agents	Mechlorethamine, melphalan, chlorambucil, cyclophosphamide, ifosfamide, carmustine (BCNU), lomustine (CCNU), mitomycin C, dacarbazine, and procarbazine	Cause reactions with different components of DNA to induce cell death	Nausea, vomiting, abdominal pain, diarrhoea, constipation, melena, stomatitis, anorexia, dry mouth, leukopenia, thrombocytopenia, encephalopathy, bone marrow suppression, and haematuria.	[28,29,30]
Targeted biological agents (cellular kinases and monoclonal antibodies)	Alemtuzumab, bevacizumab, cetuximab, gemtuzumab, ozogamicin, tiuxetan, ^131^I-tositumomab, panitumumab, rituximab, trastuzumab, bortezomib, dasatinib, erlotinib, gefitinib, imatinib, lapatinib, sorafenib, and sunitinib	Induce cell death by targeting a specific molecule in cancer cells.	Nausea, vomiting, diarrhoea, anorexia, stomatitis, abdominal pain, hepatotoxicity, cardiotoxicity, proteinuria, skin rashes, thrombosis, hypertension, myelosuppression, peripheral neuropathy, and interstitial lung disease.	[10,18]

## Data Availability

Not applicable.
